# Bronchom assuages airway hyperresponsiveness in house dust mite-induced mouse model of allergic asthma and moderates goblet cell metaplasia, sub-epithelial fibrosis along with changes in Th2 cytokines and chemokines

**DOI:** 10.3389/fimmu.2024.1384697

**Published:** 2024-05-14

**Authors:** Acharya Balkrishna, Sandeep Sinha, Shadrakbabu Karumuri, Madhulina Maity, Rishabh Dev, Anurag Varshney

**Affiliations:** ^1^Drug Discovery and Development Division, Patanjali Research Foundation, Haridwar, India; ^2^Department of Allied and Applied Sciences, University of Patanjali, Haridwar, India; ^3^Patanjali UK Trust, Glasgow, United Kingdom; ^4^Vedic Acharya Samaj Foundation, Inc., Groveland, FL, United States; ^5^Special Centre for Systems Medicine, Jawaharlal Nehru University, New Delhi, India

**Keywords:** asthma, Bronchom, house dust mite, inflammation, oxidative stress, Ayurveda

## Abstract

**Background:**

Asthma is a common obstructive airway disease with an inflammatory etiology. The main unmet need in the management of asthma is inadequate adherence to pharmacotherapy, leading to a poorly-controlled disease state, necessitating the development of novel therapies. Bronchom is a calcio-herbal formulation, which is purported to treat chronic asthma. The objective of the current study was to examine the in-vivo efficacy of Bronchom in mouse model of allergic asthma.

**Methods:**

Ultra high performance liquid chromatography was utilized to analyze the phytocompounds in Bronchom. Further, the in-vivo efficacy of Bronchom was evaluated in House dust mite (HDM)-induced allergic asthma in mice. Mice were challenged with aerosolized methacholine to assess airway hyperresponsiveness. Subsequently, inflammatory cell influx was evaluated in bronchoalveolar lavage fluid (BALF) followed by lung histology, wherein airway remodeling features were studied. Simultaneously, the levels of Th2 cytokines and chemokines in the BALF was also evaluated. Additionally, the mRNA expression of pro-inflammatory and Th2 cytokines was also assessed in the lung along with the oxidative stress markers.

**Results:**

Phytocompounds present in Bronchom included, gallic acid, protocatechuic acid, methyl gallate, rosmarinic acid, glycyrrhizin, eugenol, 6-gingerol and piperine. Bronchom effectively suppressed HDM-induced airway hyperresponsiveness along with the influx of leukocytes in the BALF. Additionally, Bronchom reduced the infiltration of inflammatory cells in the lung and it also ameliorated goblet cell metaplasia, sub-epithelial fibrosis and increase in α-smooth muscle actin. Bronchom decreased Th2 cytokines (IL-4 and IL-5) and chemokines (Eotaxin and IP-10) in the BALF. Likewise, it could also suppress the mRNA expression of pro-inflammatory cytokines (TNF-α, IFN-γ, IL-6 and IL-33), and IL-13. Moreover, Bronchom restored the HDM-induced diminution of endogenous anti-oxidants (GSH and SOD) and the increase in pro-oxidants (GSSG and MDA). Furthermore, Bronchom could also decrease the nitrosative stress by lowering the observed increase in nitrite levels.

**Conclusion:**

Taken together, the results of the present study data convincingly demonstrate that Bronchom exhibits pharmacological effects in an animal model of allergic asthma. Bronchom mitigated airway hyperresponsiveness, inflammation and airway remodeling evoked by a clinically relevant allergen and accordingly it possesses therapeutic potential for the treatment of asthma.

## 1 Introduction

Bronchial asthma is defined by “Global Initiative for Asthma (GINA)” as a heterogeneous disease, usually characterized by chronic airway inflammation. It is a common, chronic respiratory disease, whose prevalence is variable in different countries. Accordingly, as per the latest available data, asthma is estimated to affect 1-29% of the population, globally. The symptoms of asthma range from wheeze, shortness of breath, cough and tightness in the chest. These symptoms manifest due to limitation in the expiratory airflow, which is often variable. This limitation in expiratory airflow can be triggered by a host of external conditions such as exposure to allergens or irritants, exercise, weather change and viral infections affecting the respiratory system. The variable expiratory airflow obstruction may resolve spontaneously or in response to medications. Nevertheless, life threatening exacerbations of asthma are a frequently encountered, thereby resulting in a burden to both the patients and healthcare services (1).

Several clinical phenotypes of asthma have been described including allergic asthma, non-allergic asthma, adult onset asthma, asthma with persistent airflow limitation and obesity-associated asthma. Some of these phenotypes may be uncontrolled and difficult-to-treat and such patients may not even respond to conventional mainstay therapies like inhaled corticosteroids and bronchodilators. Non-adherence to inhaler-based therapeutics is a major contributor to treatment failure and consequently a major unmet need in the treatment of asthma (1). Severe asthmatics on the other hand may require biologics, which target specific inflammatory pathways, for achieving an optimal control of the disease (2). However, the sustainability of healthcare costs remains an issue with the use of biologics, as does the lack of efficacy and emergence of treatment-related adverse events (3). Moreover, affordability and availability of biologics in low and middle-income countries is a major challenge, which inevitably adds to the global burden of the disease (4). Consequently, new effective, safe, affordable and commonly accessible pharmacotherapies are required for a comprehensive management of asthma.

Ayurveda is a millennia-old, traditional form of medicinal system that largely utilizes natural herbs and minerals (5). Bronchom is an Ayurvedic medicine, which has been distinctively formulated for the treatment of obstructive airway diseases as well as interstitial lung diseases. The formulation is constituted with the extracts and powders of herbs and is additionally enriched with herbally processed minerals (**Table 1**). It is pertinent to note that the constituents of Bronchom have been therapeutically used for a long time in the Indian systems of medicine for the treatment of airway disorders.

**Table 1:** Composition of Bronchom.

S. No.	Scientific name	Vernacular name	Sanskrit Binomial name	Part used	Quantity (mg)
Dry extracts of						
**1.**	*Syzygium aromaticum* (L.) Merr. & L.M.Perry	Lavang	जम्बुक: लवङ्ग: Jambukaḥ lavagaḥ	Flower bud	10
**2.**	*Ocimum sanctum* L.	Tulsi	सुमञ्जरिका रामा Sumañjarikā rāmā	Leaf	20
**3.**	*Cinnamomum zeylanicum* Blume	Dalchini	गन्धजातकम् त्वक् Gandhajātakam tvak	Bark	10
**4.**	*Zingiber officinale* Roscoe	Sonth	आर्द्रकम् सितौष्ठम् Ārdrakam sitauṣṭham	Rhizome	10
**5.**	*Cinnamomum tamala* (Buch. -Ham.) T.Nees & C.H.Eberm.	Tejpatra	गन्धजातकम् तमालम् Gandhajātakam tamālam	Leaf	10
**6.**	*Adhatoda vasica* Nees	Safed vasa	सिंहास्यकम् वासकम् Siṃhāsyakam vāsakam	Leaf	20
**7.**	*Viola odorata* L.	Banafsa	वनप्सिका नीलपुष्पा Vanapsikā nīlapuṣpā	Flower	20
**8.**	*Clerodendrum serratum* (L.) Moon	Bharangi	प्रैकदलकम् आरीपत्रम् Praikadalakam ārīpatram	Root Bark	10
**9.**	*Glycyrrhiza glabra* L.	Mulethi	यष्टिमधुक: अरोमा Yaṣṭimadhukaḥ aromā	Root	40
**10.**	*Cassia fistula* L.	Amaltas	हेमपुष्पकम् कृतमालम् Hemapuṣpakam kṛtamālam	Fruit bulb	10
**11.**	*Piper longum* L.	Chhoti pipal	कणिका पिप्पली Kaṇikā pippalī	Fruit	10
**12.**	*Justicia gendarussa* Burm.f.	Kala vasa	सिंहास्यकम् कृष्णकाण्डम् Siṃhāsyakam kṛṣṇakāṇḍam	Leaf	20
**13.**	*Cordia dichotoma* G.Forst.	Lisoda	श्लेष्मातक: शेलु: Śleṣmātakaḥ śeluḥ	Fruit	10
**14.**	*Solanum xanthocarpum* Schrad.	Chhoti Kateli	युक्पञ्चकम् पीतप्रकण्टम् Yukpañcakam pītaprakaṇṭam	Whole plant	40
**15.**	*Datura stramonium* L.	Datura	धत्तूरक: कृष्ण: Dhattūrakaḥ kṛṣṇaḥ	Leaf	10
Fine powders of						
**16.**	*Glycyrrhiza glabra* L.	Mulethi	यष्टिमधुक: अरोमा Yaṣṭimadhukaḥ aromā	Root	32
**17.**	*Syzygium aromaticum* (L.) Merr. & L.M.Perry	Lavang	जम्बुक: लवङ्ग: Jambukaḥ lavaṅgaḥ	Flower bud	16
**18.**	*Cinnamomum zeylanicum* Blume	Dalchini	गन्धजातकम् त्वक् Gandhajātakam tvak	Bark	16
**19.**	*Pistacia integerrima* J.L.Stewart	Kakdasingi	कर्कटशृङ्गकम् रक्तपल्लवम् Karkaṭaśṛṅgakam raktapallavam	Gall	32
**20.**	*Cressa cretica* L.	Rudanti	रुदन्तक: चणपत्र: Rudantakaḥ caṇapatraḥ	Fruit	32
**21.**	*Zingiber officinale* Roscoe	Sonth	आर्द्रकम् सितौष्ठम् Ārdrakam sitauṣṭham	Rhizome	21
**22.**	*Piper longum* L.	Chhoti pipal	कणिका पिप्पली Kaṇikā pippalī	Fruit	21
**23.**	*Piper nigrum* L.	Marich	कणिका मरिचा (Kaṇikā maricā)	Fruit	21
**24.**	*Anacyclus pyrethrum* (L.) Lag.	Akarkara	आकारकरभक: छिन्नपत्र: (Ākārakarabhakaḥ chinnapatraḥ)	Root	15
**25.**	Herbally processed calcined mica ash	Abhrak Bhasma	–	Mineral	11
**26.**	Herbally processed calcined shell of Pearl oyster ash	Mukta Shukti Bhasma	–	Mineral	11
**27.**	Herbally processed calcined cowry shell ash of *Cypraea moneta* Linn.	Kapardak Bhasma	–	Mineral	11
**28.**	Herbally processed calcium rich gypsum ash	Godanti Bhasma	–	Mineral	11

Herbo-mineral constituents of Bronchom have been described for their therapeutic utility in respiratory diseases, in the Ayurvedic reference texts namely, Bhavprakash Nighantu, Edition-2006 and 2010 (S.No. 1-23); The Ayurvedic Pharmacopoeia of India, Part-1, Volume-II (S.No, 24); Ayurved Sar Sangrah, Edition-2010 (S.No. 25-27) and The Ayurvedic Formulary of India-I, 2^nd^ Edition (S. No. 28). Gum acacia, Talcum, Microcrystalline cellulose and Croscarmellose sodium have been used for formulating the Bronchom tablet.

The objective of the current study was to evaluate the efficacy of Bronchom in mouse model of house dust mite (HDM)-induced allergic asthma. HDM was specifically chosen to elicit the disease induction as it is a relevant causative agent for the development of asthma in humans (6). In the current study, Bronchom was administered prophylactically, 15-days prior to disease induction followed by instillation of HDM for five consecutive weeks. Thereafter, airway hyperresponsiveness to aerosolized methacholine was assessed. Methacholine is a non-selective muscarinic receptor agonist, which acts on the receptors present on the airway smooth muscle to elicit bronchoconstriction (7). It is a non-specific bronchial spasmogen, which has been reported to exhibit markedly enhanced bronchoconstriction in HDM-challenged mice when compared to unchallenged animals (8). After completion of the assessment of airway hyperresponsiveness, the bronchoalveolar lavage fluid was collected for cytological analysis as well as for the estimation of cytokines and chemokines. Subsequently, lungs were harvested for histological, biochemical and gene expression analysis. In addition to in-vivo efficacy assessment, the phytochemical analysis of Bronchom was also performed to identify potential bioactive marker compounds, with an objective of explicating the observations of the in-vivo experiment.

## 2 Materials and methods

### 2.1 Test article, chemicals and reagents

Bronchom (Internal batch no: CHIH/BROA/0421/1128) was sourced from Divya Pharmacy, Haridwar, India. The phyto- and mineral components of the test article are mentioned in **Table 1**. Dexamethasone (D4902), Geimsa stain (48900), gallic acid (91215; potency-97.3%), eugenol (35995; potency-99.6%), piperine (P49007; potency-97.0%), rosmarinic acid (CFN99103; potency-98.0%) and anti-Actin, α-Smooth Muscle antibody (A5228) were purchased from Sigma-Aldrich, USA. Methyl gallate (G0017; potency-99.9%), methacholine (M0073) and succinylcholine (S0149) were procured Tokyo Chemical Industries, India. Protocatechuic acid (P006; potency-99.5%) and glycyrrhizin (G2137; potency-93.0%) were bought from Natural Remedies), whereas 6-gingerol (11707; potency-99.3%) was procured from Cayman Chemical. House dust mite, comprised of the crushed whole bodies of *Dermatophagoides pteronyssinus* (XPB70D3A2.5) was purchased from Stallergenes Greer, USA. Thiopental sodium was bought from Neon Laboratories Limited, India whereas isoflurane was purchased from Baxter, India. The powder for preparing Hank’s Balanced Salt Solution (HBSS, TS1098) was obtained from HiMedia, India. Th2 cytokines and chemokines in the BALF were evaluated by employing multiplexing kit (MCYTOMAG-70K-08) and was purchased from Merck. TRIzol reagent (15596018), Verso cDNA synthesis kit (AB-1453/B), Alexa Fluor Plus 488 (A32723) and DAPI (D1306, 4’,6-Diamidino-2-Phenylindole, dihydrochloride) were purchased from Thermo Fisher Scientific, USA.

### 2.2 Ultra high performance liquid chromatography-photodiode array analysis of Bronchom

To 250 mg of Bronchom powder, 5 mL of water: methanol (20:80) was added. It was then sonicated for 30 min, centrifuged at 10000 rpm for 5 min and finally filtered using a 0.45 µm nylon filter. This solution was used for the further analysis. The stock solutions of gallic acid, methyl gallate, protocatechuic acid, eugenol, 6-gingerol, piperine, rosmarinic acid and glycyrrhizin were dissolved in methanol to prepare standard solutions of concentration 1000 µg/mL, individually. Subsequently, standard mix working solution of 50 µg/mL concentration were prepared for each of the standards. Analysis was performed on a Prominence-XR UHPLC system (Shimadzu, Japan), which was equipped with Quaternary pump (NexeraXR LC-20AD XR), PDA detector (SPD-M20 A), autosampler (Nexera XR SIL-20 AC XR), degassing unit (DGU-20A 5R) and column oven (CTO-10 AS VP). Separation by UHPLC was achieved using a Shodex C18-4E (5µm, 4.6 × 250 mm) column subjected to binary gradient elution. The two solvents used for the analysis consisted of water containing 0.1% orthophosphoric acid; adjusted to pH 2.5 with diethyl amine (solvent A) and acetonitrile (solvent B). Gradient programming of the solvent system was initially at 5% B for 0-5 min, 5-25% B from 5-30 min, 25-45% B from 30-40 min, 45-70% B from 40-50 min, 70% B from 50-55 min, 70-85% B from 55-65 min, 85-5% B from 65-66 min, 5% B from 66-70 min with a flow rate of 1.0 ml/min. 10 µl of standard and sample solution were injected and column temperature was maintain at 35°C. Wavelengths were set 270 nm (for gallic acid, methyl gallate, protocatechuic acid, eugenol and 6-gingerol), 250 nm (for glycyrrhizin) and 325 nm (for rosmarinic acid and piperine).

### 2.3 Experimental animals and husbandry

Female, specific pathogen free BALB/c mice (6-7 weeks of age) were purchased from Hylasco Biotechnology (India) Pvt. Ltd., Telangana, India, a Charles River Laboratories-accredited domestic animal breeder. The current study is reported according to the ARRIVE guidelines (9) and all the experimental procedures performed were in strict accordance with the recommendations specified by Committee for Control and Supervision of Experiments on Animals, Department of Animal Husbandry and Dairying, Ministry of Fisheries, Animal Husbandry and Dairying, Government of India. Prior to the initiation of the experiments, the experimental protocol was thoroughly reviewed and subsequently ratified by the Institutional Animal Ethics Committee of Patanjali Research Foundation, vide protocol number PRIAS/LAF/IAEC-103. Post-receipt, the mice were quarantined for a week and were then shifted to an experimental room, earmarked for the study. Here the animals were housed in polypropylene cages of standard dimensions at room temperature ranging from of 21-25 °C and relative humidity between 60-70%, along with a 12-hour light/dark cycle. Animals had unrestricted supply of gamma-irradiated standard pelleted laboratory animal diet (Purina 5L79 Rodent Lab diet, USA), which was purchased from Hylasco Biotechnology (India) Pvt. Ltd., Telangana, India. Similarly, reverse osmosis (Make: MERCK) water was also provided *ad libitum* in steam sterilized polypropylene bottles. Due care was taken to minimize animal suffering during the entire course of the study. Intranasal instillation of HDM was performed under transient isoflurane anesthesia. Further, lung function assessment was performed under thiopental sodium anesthesia (50 mg/kg), administered intraperitoneally and at the end of the experiment the mice were humanely sacrificed under overdose of thiopental sodium anesthesia (150 mg/kg), which was also administered by the intraperitoneal route.

### 2.4 Calculation of the doses for the *in-vivo* study

The doses of Bronchom tested in the present study were computed on the basis of the variances in the body surface areas of humans and mice. The clinically recommended therapeutic dose of Bronchom is 2000 mg/day, in two divided doses of 1000 mg, respectively. Accordingly, the therapeutic dose for a human weighing 60 kg will be 2000/60 i.e. 33.33 mg/kg/day. The equivalent dose (in mg/kg) for mice was computed by multiplying the human equivalent dose (in mg/kg) by factor of 12.3 (10). The mouse equivalent dose was consequently calculated to be 409.99 mg/kg/day. Rounding off to the nearest hundreds, 400 mg/kg/day or 200 mg/kg, b.i.d. was adjudged to be the mouse equivalent dose. With an objective of capturing a dose-response relationship in the studied parameters, the remaining doses chosen were from 1/10^th^ to 3 times the recommended therapeutic dose i.e. 40, 120 and 1200 mg/kg/day. This corresponds to twice daily doses of 20, 60 and 600 mg/kg, respectively.

### 2.5 Administration of the compounds and development of the mouse model of allergic asthma

**Figure 1:**
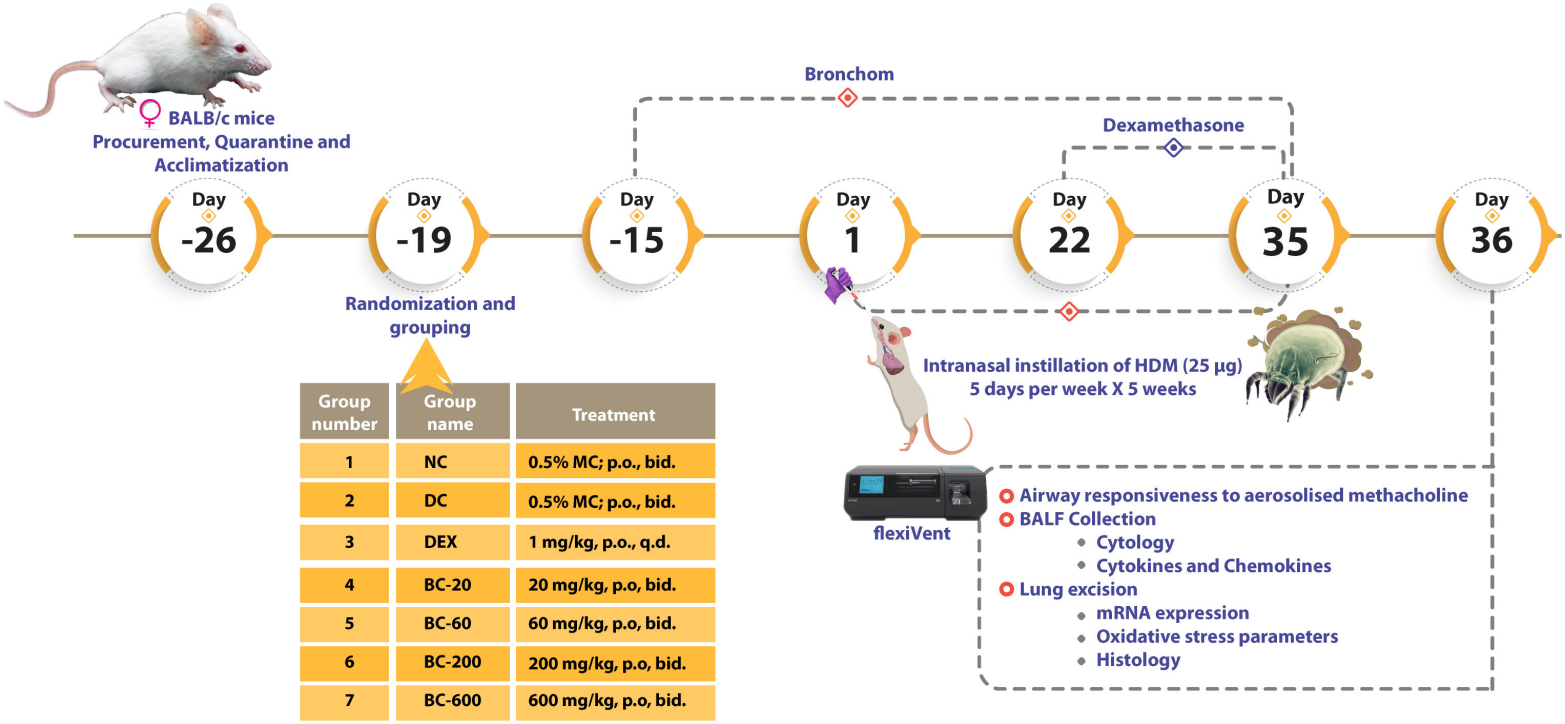
Schematic representation of the evaluation of Bronchom in mouse model of house dust mite-induced allergic asthma. After completion of quarantine and acclimatization, female BALB/c mice were administered Bronchom (BC), prophylactically daily for 15-consecutive days. During this experimental period, mice allocated to normal-control (NC), disease-control (DC) and dexamethasone (DEX)-treated group were administered 0.5% methylcellulose (MC) orally, twice daily. The animals that received Bronchom were administered the formulation as a suspension in 0.5% MC by gavage twice daily, in escalating doses of 20, 60, 200 and 600 mg/kg, b.i.d. After completion of the prophylactic administration, animals were intranasally instilled with 25 µg of house dust mite (HDM) protein (dissolved in 20 µL of saline), five days a week for five consecutive weeks. The dexamethasone treated group started receiving the compound three weeks after initiation of disease induction at the dose of 1 mg/kg, once daily by oral route. Twenty-four hours after the last instillation, animals were anaesthetized and airway responsiveness to aerosolized methacholine was evaluated. Subsequently, the animals were euthanized and bronchoalveolar lavage fluid (BALF) was collected for estimation of the influx of inflammatory cells as well as for the determination of cytokines and chemokines. Finally, the bilateral lungs of the animals were excised. Histological assessments were conducted in the left lung, whereas the right lung was preserved for mRNA expression studies of pro-inflammatory cytokines and a Th2 cytokine, as well as for the evaluation of oxidative stress.

After completion of quarantine, mice were randomized on the basis of their respective body weights and subsequently allocated into seven groups as outlined in **Figure 1**.

Bronchom was daily administered to the mice, prophylactically as a gavage, for 15-consecutive days prior to initiation of disease induction, whereas dexamethasone was administered 3 weeks after commencement of disease induction, orally. The dose volume was fixed at 10 mL/kg and 0.5% methylcellulose (MC) solution was used as the vehicle for formulating the compounds.

To induce allergic asthma, 25 µg of HDM protein dissolved in 20 µL of normal saline was instilled into the nares of the mice, 5 days a week for 5-consecutive weeks, under transient isoflurane anesthesia. Animals allocated to Group 1 were administered 20 µL of normal saline, intranasally Further, the animals continued to receive their respective treatments, as outlined above, for the entire duration of the experiment, concurrent with HDM instillation.

### 2.6 Measurement of airway responses to aerosolised methacholine

Twenty-four hours after the last HDM instillation, the mice were anaesthetized by intraperitoneal administration of thiopental sodium (50 mg/kg). They were then tracheostomized, followed by the insertion of an 18-gauge stainless steel cannula in the trachea. Subsequently, respiratory paralysis was induced by intraperitoneal administration of succinylcholine-7.5 mg/kg following which, the tracheal cannula was attached to a flexiVent system (Emka-Scireq, Canada). The intubated mice were ventilated with a tidal volume of 10 mL/kg at 150 breaths per minute, with a positive end expiratory pressure of 3 cm of H_2_O. The animals were then subjected to challenge with escalating doses of aerosolized methacholine, ranging from 0 (saline) to 50 mg/mL. By employing the low frequency forced oscillation technique, total respiratory system resistance (Rrs) and total respiratory system elastance (Ers) was derived from the single compartment model. The Rrs and Ers values obtained subsequent to challenging the animals allocated to groups 2 to group 7, at the concentration of 50 mg/mL of methacholine, was then normalized with the response obtained in the normal-control group (Group 1) at 50 mg/mL. Average value of Rrs or Ers obtained at MCh-50 mg/mL in Group 2 (disease-control group) was utilized for the calculation of % protection from AHR. The percentage protection from airway hyperresponsiveness (AHR) for Groups 3-7 was then computed by employing the following formula:


$$\%\;Protection\;from\;AHR\;=\;\frac{Normalized\;Rrs\;\left(or\;Ers\right)\;in\;Group\;2-Normalized\;Rrs\;\left(or\;Ers\right)\;in\;Treated\;groups\;}{Normalized\;Rrs\;\left(or\;Ers\right)\;in\;Group\;2}\;\times 100$$

### 2.7 Assessment of the cellularity of BALF

After the measurement of lung mechanics, the animals were euthanized with thiopentone administered intraperitoneally at the dose of 150 mg/kg. Then 1 ml of ice cold HBSS was slowly introduced into the lungs via the tracheal cannula. Subsequently, the thorax of the animal was gently massaged and the BALF was collected in a centrifuge tube. This exercise was repeated for four additional times. The first BALF retrieved from the animals was centrifuged at 2800 × g for 10 minutes at 4°C by employing a refrigerated centrifuge (Sorvall Legend Micro 21R; Thermo Fisher Scientific Inc., USA). Subsequently, the supernatant was separated and immediately stored at -80 °C for cytokine estimation. The cell pellet was then re-suspended with 1 ml of HBSS. The remaining lung washes were similarly centrifuged using a refrigerated centrifuge (Sorvall ST 8R; Thermo Fisher Scientific Inc.; USA) and the retrieved cell pellet was then pooled with the one obtained from the first wash. Total leukocytes in the re-suspended cell pellet were enumerated by employing a hematology analyzer (BC5000 Vet, Mindray, China). Further, the differential leukocyte counts were enumerated in smears obtained by employing a cytocentrifuge (Medspin5; Medilabsolutions; India), set at 500 rpm for 10 min. The obtained smears were then stained with Giemsa stain. A total of three hundred leukocytes were counted by using a microscope (Olympus BX43, Perkin Elmer; USA) and the following cells were identified in the stained smears according to their morphology: macrophages, eosinophils, lymphocytes, neutrophils and basophils. Subsequently, the counted cells were expressed as a percentage and the absolute counts of each of the identified leukocytes were computed by employing the formula.


Absolute cell count=% of the identified leukocytes ×Total cell count

### 2.8 Harvesting of lungs and processing for histopathology and immunofluorescence

After completion of BALF collection, lungs were excised from the animals. The left lung was fixed in 10% neutral buffered formalin and the right lung was flash frozen in liquid nitrogen and stored at -80 °C for biochemical and molecular estimations. The left lung was processed for histopathological evaluation using standard procedures and subsequently 3-5 µm sections were stained with hematoxylin and eosin (H&E) stain to detect the manifestation of infiltration of inflammatory cells, Periodic Acid-Schiff (PAS) stain for evidence of goblet cell metaplasia and Masson’s Trichrome (MT) stain for the presence of sub-epithelial fibrosis, as described previously (5). In order to assess the severity of peribronchiolar and perivascular inflammation as well as sub-epithelial fibrosis, the following semi-quantitative scoring system was employed: 0 = Nil; 1 = Mild; 2 = Moderate and 3 = Severe. Further, to assess the extent of goblet cell metaplasia, the following scoring criteria was used, based on the number of goblet cells present per bronchiole: 0 = 0-75 cells; 1 = 76-150 cells; 2 = 151-225 cells; 3 = 226-300 cells; 4 = 301-375 cells; 5 = 376-450 cells; 6 = 451-525 cells; 7 = 526-600 cells; 8 = 601-675 cells and 9 = 676-750 cells.

Additionally, the increase in airway smooth muscle mass was determined by morphometric analysis subsequent to immunofluorescence staining of alpha-smooth muscle actin (α-SMA). For this procedure, processed lung tissues were cut in 2-2.5 micron sections and mounted on poly-l-lysine coated slides. Then the sections were deparaffinized in xylene and rehydrated in descending grades of alcohol. Finally, these sections were washed in deionized water twice. For antigen retrieval, sections were subjected to heat-induced epitope retrieval using sodium citrate buffer (pH 6.0). The slides were cooled followed by washing in deionized water and blocking of non-specific binding using blocking buffer (Vitro Master Diagnostica Master polymer plus detection system). Then the sections were immunolabelled with primary antibody (Anti-Actin, α-Smooth Muscle antibody, 1:2000 dilution) and incubated at room temperature for 1 hour followed by staining with secondary antibody (Alexa Fluor 488, 1:500 dilution) at room temperature for 1 hour. The sections were washed in PBS followed by staining with DAPI with anti-fade mountant and mounted with coverslip. Then fluorescent evaluation was performed using an Olympus BX43 fluorescent microscope and images were obtained using Mantra Imaging System (PerkinElmer). The thickness of alpha smooth muscle actin around the bronchioles was measured by employing Zeiss Axioscope microscope, using AxioVision software, in six randomly chosen areas per animal. The average of the measured thickness was subsequently computed for each animal.

### 2.9 Measurement of cytokines and chemokines in the BALF

The cytokines (IL-4 and IL-5) and chemokines (Eotaxin and IP-10) were measured by multiplexing that employed a MILLIPLEX^®^MAP Mouse Cytokine/Chemokine Magnetic Bead Panel based on the Luminex^®^ xMAP^®^ technology (Merck).

### 2.10 Quantitative real-time PCR for mRNA expression in lung

The total RNA was extracted from lung tissue using TRIzol reagent and was used to synthesize the cDNA. 1μg of the total RNA was used with Verso cDNA synthesis kit. The qRT-PCR analysis was performed as described previously (11), using GAPDH as housekeeping gene. Primers used are mentioned in **Supplementary Table 1**.

### 2.11 Determination of oxidative and nitrosative stress parameters in the lung tissue

Levels of reduced glutathione (GSH) and oxidized Glutathione (GSSG) were estimated as per previously reported methods (12). Superoxide dismutase (SOD) activity was determined as per the method of Beauchamp and Fridovich, 1971 (13), whereas malondialdehye (MDA) activity was measured according to the method of Heath and Packer, 1968 (14). For nitrosative stress, nitrite levels were measured as previously reported (15). The values were normalized to the protein content of the lung, which was additionally estimated in the lung homogenate by Bicinchoninic acid (BCA) assay using a commercially available kit (Thermo Fisher), as per the manufacturer’s instructions.

### 2.12 Statistical analysis

All the data for the studied parameters were compiled from each of the study groups and expressed as mean ± standard error of mean (SEM). Statistical analysis was performed using GraphPad Prism version 7.04 software (GraphPad Software, San Diego, CA, USA). A one-way analysis of variance (ANOVA) followed by Dunnett’s multiple comparison post-hoc test was employed to calculate the statistical differences between the mean values for all the evaluated parameters except AHR measurement at different concentration of MCh, for which, two-way ANOVA followed by Tukey’s multiple comparison test was employed. A p value < 0.05 was considered to be statistically significant.

## 3 Results

### 3.1 Bronchom is phytochemically enriched with bioactive compounds known to exert a beneficial effect in allergic asthma

**Figure 2:**
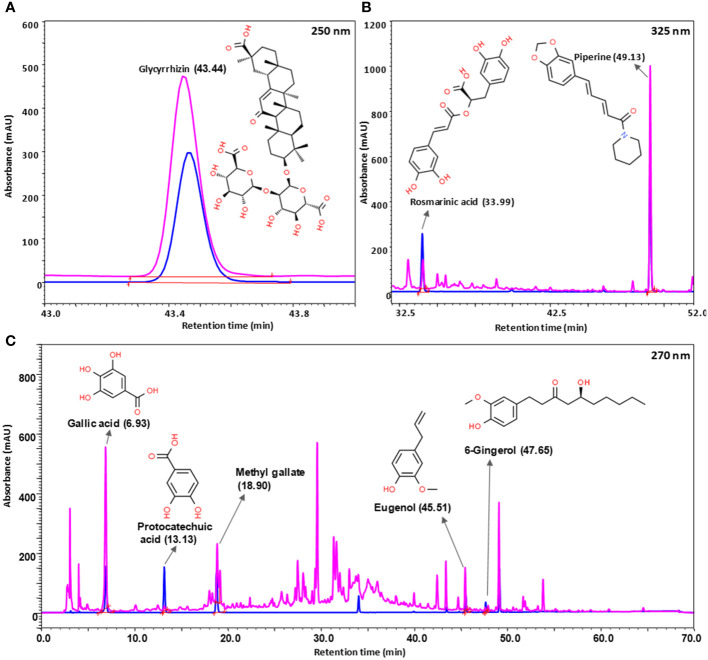
Phytochemical analysis of Bronchom. Bronchom was subjected to analysis on UHPLC-PDA platform (chromatogram depicted in pink) and compared to pure reference standards (chromatogram represented in blue). Eight compounds were identified and quantified in Bronchom as summarized in **Table 2**. The presence of glycyrrhizin was detected at 250 nm **(A)**; rosmarinic acid and piperine were identified at 325 nm **(B)**, whereas the remaining five compounds, namely gallic acid, protocatechuic acid, methyl gallate, eugenol and 6-gingerol were detected at 270 nm **(C)**. The chemical structures of the identified phytochemicals have been incorporated along with the chromatograms, and have been obtained from www.chemspider.com.

UHPLC-PDA analysis of Bronchom revealed the presence of eight bioactive phyto-metabolites, subsequent to comparison with the obtained chromatographs of pure standard compounds (**Table 2** and **Figure 2**). Each milligram of Bronchom powder contained gallic acid (3.407 µg), protocatechuic acid (0.092 µg), methyl gallate (1.393 µg), rosmarinic acid (0.793 µg), glycyrrhizin (8.281 µg), eugenol (2.332 µg), 6-gingerol (0.308 µg) and piperine (2.411 µg).

**Table 2:** Phytocompounds identified and quantified in Bronchom by UHPLC-PDA analysis, as depicted in **Figure 2**.

S. No.	Phytocompound detected	Retention Time (minutes)	Content in Bronchom (µg/mg)
1	Gallic acid	6.93	3.407
2	Protocatechuic acid	13.13	0.092
3	Methyl gallate	18.90	1.393
4	Rosmarinic acid	33.99	0.793
5	Glycyrrhizin	43.44	8.281
6	Eugenol	45.51	2.332
7	6-Gingerol	47.65	0.308
8	Piperine	49.13	2.411

### 3.2 Bronchom reduces the development of HDM-induced airway hyperresponsiveness to aerosolized methacholine

**Figure 3:**
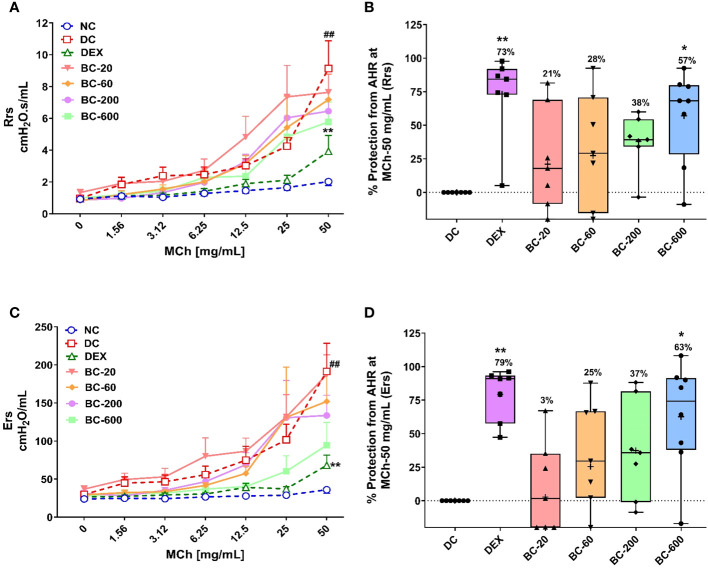
Bronchom limits the development of HDM-induced AHR in a dose-related manner. Mice were challenged with escalating doses of Methacholine (MCh), twenty-four hours after the final HDM-instillation as elaborated in the Materials and Methods section. **(A)** Represents the total respiratory system resistance (Rrs) values obtained subsequent to MCh challenge at concentrations ranging from 0 to 50 mg/mL. **(B)** Represents the percentage protection from HDM-induced AHR and was calculated by normalizing the Rrs values in animals allocated to groups 2 to group 7, obtained after challenging the mice with MCh-50 mg/mL, with the Rrs values correspondingly observed in the normal-control group. Values mentioned above the whisker plots denote percentage protection. **(C)** Represents total respiratory system elastance (Ers) values obtained after MCh challenge with concentrations ranging from 0 to 50 mg/mL. **(D)** Represents the percentage protection from HDM-induced AHR and was calculated by normalizing the Ers values in animals allocated to groups 2 to group 7, obtained after challenging the mice with MCh-50 mg/mL, with the Ers values correspondingly observed in the normal-control group. Values mentioned above the whisker plots indicate percentage protection. The average Rrs and Ers values in **(B, D)**, respectively have been denoted with the + symbol. Data is presented as mean ± S.E.M (n=7-8 animals per group). For the data depicted in **(A, C)**, analysis was performed by employing two-way ANOVA followed by Tukey’s multiple comparison test, whereas the data depicted in **(B, D)** was analyzed by one-way ANOVA followed by Dunnett’s multi-comparison *post hoc* test. ##; p < 0.01 vs. saline-challenged (Normal-control) group. *p < 0.05; **p < 0.01 vs. HDM challenged (Disease-control group).

Repeated intranasal exposure of HDM to mice for five weeks resulted in an increased bronchoconstrictor response to aerosolized methacholine, reflected by an increase in the Rrs and Ers values, when compared to the normal-control group (**Figures 3A–D**). A significant difference between the airway responsiveness of disease control *vs.* normal control animals was evident when they were challenged with the highest concentration of methacholine-50 mg/mL (**Figures 3A, C**). Hence, we normalized the increased Rrs and Ers observed in disease control animal with that noted in the normal-control animals and accordingly computed the percentage protection from AHR for all the treated groups. Reference control dexamethasone administered at the dose of 1 mg/kg, p.o., significantly reversed HDM-induced AHR (**Figures 3B, D**), thereby validating the model. Bronchom administered orally at the doses of 20, 60, 200 and 600 mg/kg, b.i.d. reduced the development of AHR in a dose-related manner (**Figures 3B, D**). Significant reduction of AHR was evident at the highest tested dose of Bronchom.

### 3.3 Bronchom reduces the HDM-induced influx of inflammatory cells in the BALF

**Figure 4:**
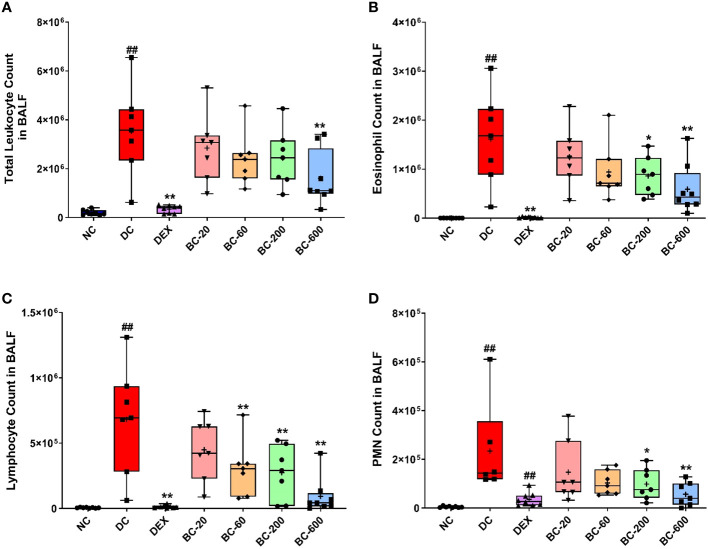
Bronchom reduces HDM-induced inflammatory cell influx in BALF. Animals were sacrificed after measurement of AHR and BALF was collected as mentioned in the Materials and Methods section to enumerate **(A)** Total leukocyte count; **(B)** Eosinophil count; **(C)** Lymphocyte count and **(D)** Polymorphonuclear (PMN) cell or neutrophil count. Data is presented as Mean ± S.E.M (n=7-8 animals per group). The average value for each of the whisker plots has been denoted by the + symbol. Data was analyzed by one-way ANOVA followed by Dunnett’s multi-comparison *post hoc* test. ##; p < 0.01 vs. saline-challenged (normal-control) group. *, p < 0.05; **, p < 0.01 vs. HDM challenged (Disease-control group).

Similar, to the development AHR, repeated HDM-challenge in untreated animals, elicited a robust increase in BALF cellularity as evident by a significant elevation in the number of total leukocytes, eosinophils, lymphocytes and neutrophils, as compared to the normal-control animals (**Figures 4A–D**). Dexamethasone, completely abrogated the HDM-induced infiltration of these inflammatory cells in the BALF. Oral administration of Bronchom at the tested doses, exhibited a dose-related inhibition of HDM-induced influx of inflammatory cells in the BALF (**Figures 4A–D**). When compared to the disease-control group, Bronchom-600 mg/kg, b.i.d. significantly inhibited the infiltration of the total inflammatory cells and neutrophils. Further, the significant inhibitory effects of Bronchom on eosinophil influx were evident at the doses of 200 and 600 mg/kg, b.i.d., respectively. The most pronounced effect of Bronchom was observed in the inhibition of absolute lymphocyte influx, wherein, significant differences were noted at the doses of 60, 200 and 600 mg/kg, b.i.d., respectively.

### 3.4 Bronchom alleviates HDM-induced features of airway remodeling

**Figure 5:**
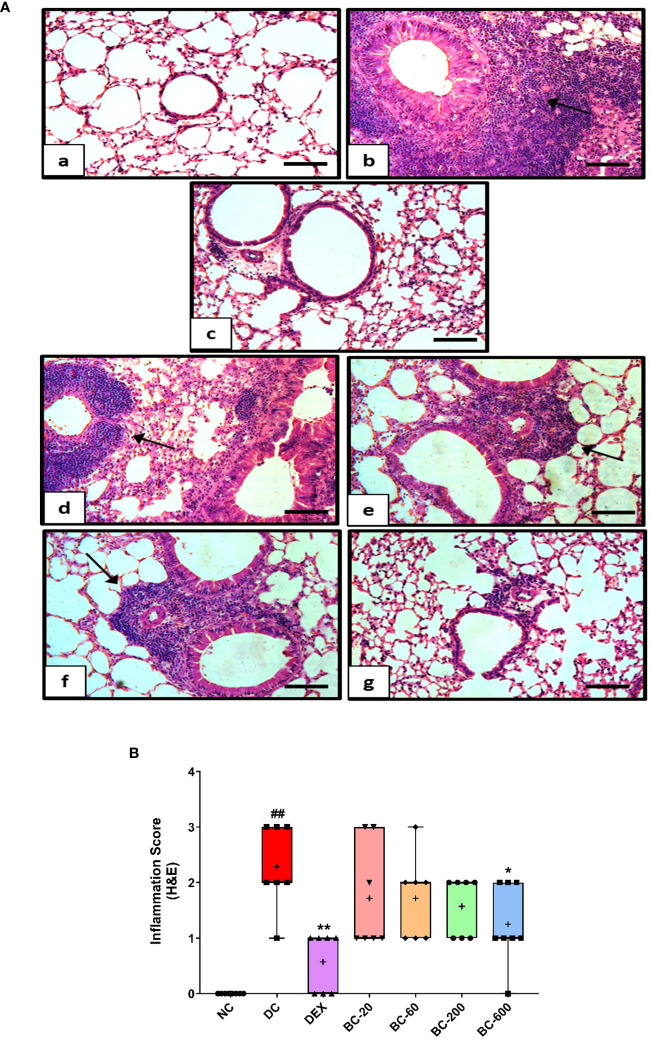
Bronchom decreases HDM-induced peribronchiolar and perivascular inflammation. Lungs were harvested from animals and processed for Haematoxylin and Eosin (H&E) staining. **(A)** Representative photomicrographs of the lung × 100 from a) Normal-control group b) Disease-control group c) Dexamethasone-1 mg/kg, once daily d) Bronchom-20 mg/kg, b.i.d. e) Bronchom-60 mg/kg, b.i.d. f) Bronchom-200 mg/kg, b.i.d. and g) Bronchom-600 mg/kg, b.i.d. Scale bar = 100 µm. Arrow indicates peribronchiolar and perivascular inflammation. **(B)** Semi-quantitative lung inflammation score. Data is presented as mean ± S.E.M (n=7-8 animals per group). The average value for each of the whisker plots has been denoted by the + symbol. Data was analyzed by one-way ANOVA followed by Dunnett’s multi-comparison *post hoc* test. ##; p < 0.01 vs. saline-challenged (Normal-control) group. *, p < 0.05; **, p < 0.01 vs. HDM challenged (Disease-control group).

**Figure 6:**
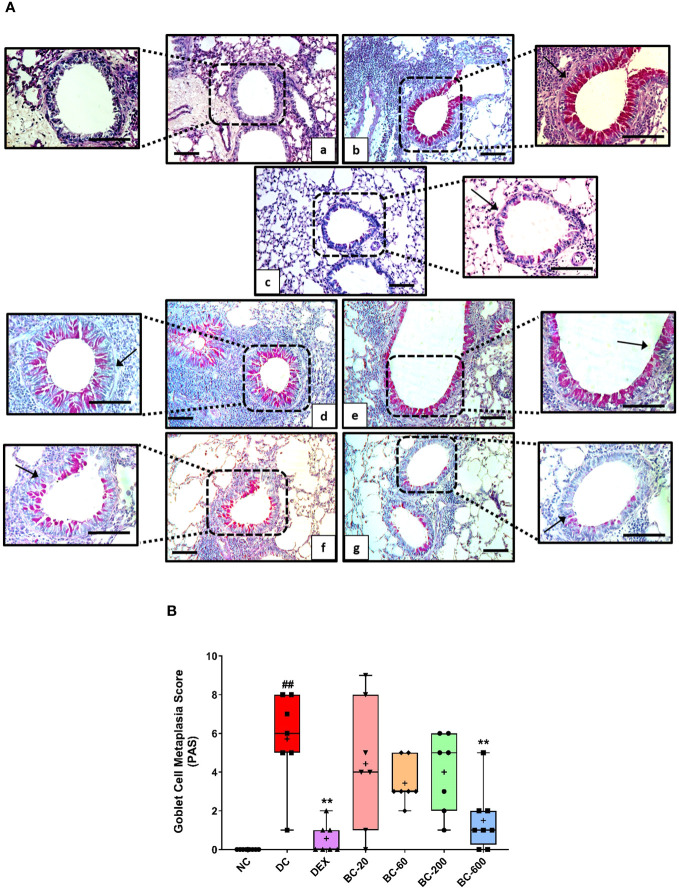
Bronchom attenuates HDM-induced goblet cell metaplasia. Lungs were excised from animals and processed for Periodic acid–Schiff staining (PAS). **(A)** Representative stained sections of the lung × 100 and × 200 from a) Normal-control group b) Disease-control group c) Dexamethasone-1 mg/kg, once daily d) Bronchom-20 mg/kg, b.i.d. e) Bronchom-60 mg/kg, b.i.d. f) Bronchom-200 mg/kg, b.i.d. and g) Bronchom-600 mg/kg, b.i.d. Scale bar = 100 µm. Arrow indicates PAS positive goblet cells. The photomicrographs where the black boxes are depicted are acquired at 100 times magnification. In addition, the field within the black boxes has been acquired at 200 times magnification and has been placed adjacent to the lower magnification photomicrograph. **(B)** Semi-quantitative goblet cell metaplasia score. Data is presented as mean ± S.E.M (n=7-8 animals per group). The average value for each of the whisker plots has been denoted by the + symbol. Data was analyzed by one-way ANOVA followed by Dunnett’s multi-comparison *post hoc* test. ##; p < 0.01 vs. saline-challenged (Normal-control) group. **, p < 0.01 vs. HDM challenged (Disease-control group).

**Figure 7:**
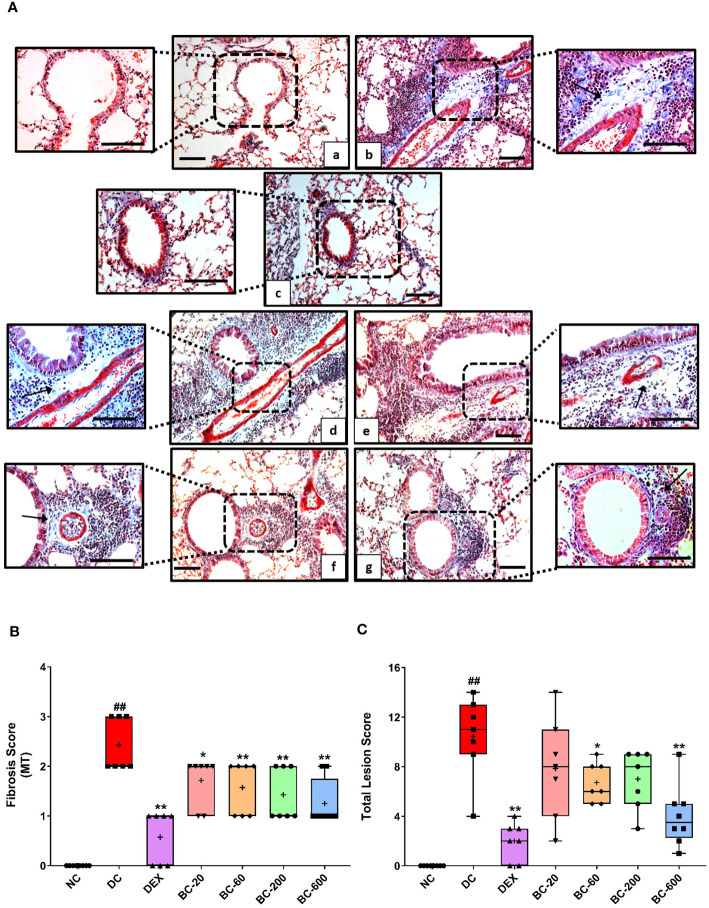
Bronchom attenuates HDM-induced sub-epithelial fibrosis. Lungs were harvested from animals and processed for Masson’s Trichrome (MT) staining. **(A)** Representative photomicrographs of the lung × 100 and × 200 from a) Normal-control group b) Disease-control group c) Dexamethasone-1 mg/kg, once daily d) Bronchom-20 mg/kg, b.i.d. e) Bronchom-60 mg/kg, b.i.d. f) Bronchom-200 mg/kg, b.i.d. and g) Bronchom-600 mg/kg, b.i.d. Scale bar = 100 µm. Arrow indicates collagen positive area. The photomicrographs where the black boxes are depicted are acquired at 100 times magnification. The field within the black boxes has been acquired at 200 times magnification and has been placed adjacent to the lower magnification photomicrograph. **(B)** Semi-quantitative sub epithelial fibrosis score. **(C)** Semi-quantitative total lesion score. Data is presented as mean ± S.E.M (n=7-8 animals per group). The average value for each of the whisker plots has been denoted by the + symbol. Data was analyzed by one-way ANOVA followed by Dunnett’s multi-comparison *post hoc* test. ##; p < 0.01 vs saline-challenged (normal-control) group. *, p < 0.05; **, p < 0.01 vs. HDM-challenged (Disease-control group).

**Figure 8:**
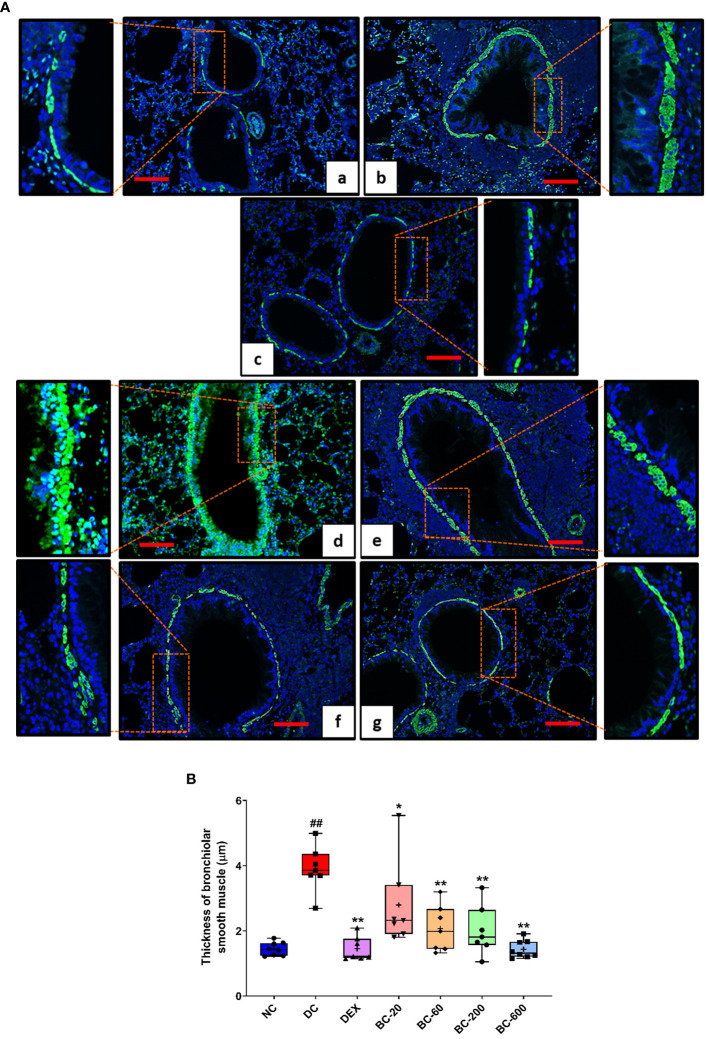
Bronchom reduces HDM-induced increase in airway smooth muscle mass. Lungs were harvested from the animals and processed for alpha-smooth muscle actin imaging using the immunofluorescence technique as described in the Material and Methods section. **(A)** Representative sections of the lung at × 100 from a) Normal-control group b) Disease-control group c) Dexamethasone-1 mg/kg, once daily. d) Bronchom-20 mg/kg, b.i.d. e) Bronchom-60 mg/kg, b.i.d. f) Bronchom-200 mg/kg, b.i.d. and g) Bronchom-600 mg/kg, b.i.d. The photomicrographs where the red rectangular boxes are depicted are acquired at 100 times magnification. In addition, the field within the red boxes has been digitally zoomed by four times for a greater clarity of the stained airway smooth muscle. Scale bar = 100 µm. **(B)** Thickness of alpha-smooth actin in the sections. Data is presented as Mean ± S.E.M (n=7-8 animals per group) The average value for each of the whisker plots has been denoted by the + symbol. The data was analyzed by one-way ANOVA followed by Dunnett’s multi-comparison *post hoc* test. ##; p < 0.01 vs. saline-challenged (Normal-control) group. *, p < 0.05; **, p < 0.01 vs. HDM-challenged (Disease-control) group.

When compared to the saline-challenged animals, all the features of airway remodeling were clearly evident in mice that were subjected to repeated HDM exposure. These included peri-bronchiolar and peri-vascular inflammation (**Figure 5**), goblet cell metaplasia (**Figure 6**), sub-epithelial fibrosis (**Figure 7**), and increased alpha-smooth muscle actin around the airways (**Figure 8**). The reference control drug, dexamethasone ameliorated the airway remodeling changes. Oral administration of Bronchom inhibited inflammatory cell infiltration in the peri-bronchial and peri-vascular areas in a dose-related manner (**Figures 5A, B**) with a significant inhibition observed at the dose of 600 mg/kg, b.i.d. Further, the highest tested dose of Bronchom was able to significantly limit the development of goblet cell metaplasia (**Figures 6A, B**). Additionally, Bronchom reduced the emergence of HDM-induced sub-epithelial fibrosis (**Figures 7A, B**), wherein, significant effects were evident at all the tested doses of Bronchom. When compared to the disease-control group, the total histopathological lesion score was significantly lesser in Bronchom-60 and 600 mg/kg, b.i.d. treated groups, respectively.

Immunofluorescence staining of alpha-smooth muscle actin in the sections of the lung additionally revealed that Bronchom was able to dose-dependently reduce the HDM-induced increase in the airway smooth muscle mass (**Figures 8A, B**) and the effects were significant at all the tested doses.

### 3.5 Bronchom reduces the HDM-induced release of Th2 cytokines and chemokines in the BALF

**Figure 9:**
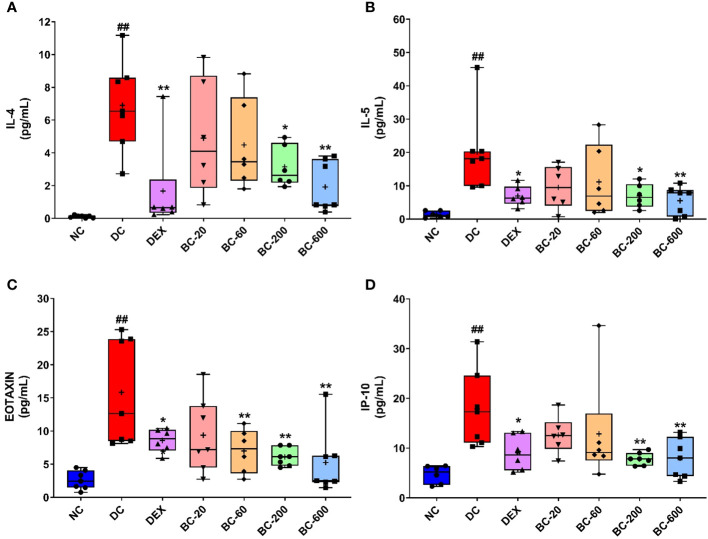
Bronchom reduced HDM-induced release of Th2 cytokines and chemokines in BALF. Selected Th2 cytokines and chemokines were analyzed in the BALF by multiplexing as elaborated in the in the Material and Methods section and expressed in pg/mL. **(A)** IL-4, **(B)** IL-5 **(C)** Eotaxin and **(D)** IP-10. Data is presented as Mean ± S.E.M (n=6-7 animals per group). The average value for each of the whisker plots has been denoted by the + symbol. The data was analyzed by one-way ANOVA followed by Dunnett’s multi-comparison *post hoc* test. ##, p < 0.01 vs. saline-challenged (Normal-control) group. *, p < 0.05, **, p < 0.01 vs. HDM-challenged (Disease-control group).

HDM exposure in mice for 5-weeks resulted in significant elevation of Th2-cytokines, IL-4 and IL-5 as well as chemokines (Eotaxin and IP-10) in BALF (**Figures 9A–D**). Dexamethasone-1 mg/kg, p.o. significantly decreased (**Figures 9A–D**) the induction of the evaluated cytokines and chemokines. Oral administration of Bronchom exhibited a dose-related inhibition of HDM-induced increase in the levels of the tested cytokines and chemokines (**Figures 9A–D**). For IL-4 and IL-5 the effect was significant at 200 and 600 mg/kg, b.i.d. as compared to the disease-control group. Further, for Eotaxin the effect was statistically significant at 60, 200 and 600 mg/kg, b.i.d., respectively. Furthermore, in the case of IP-10, the effect was statistically significant, when compared to the disease-control group at 200 and 600 mg/kg, b.i.d.

### 3.6 Bronchom moderates HDM-induced mRNA expression of pro-inflammatory and a Th2 cytokine in the lungs

**Figure 10:**
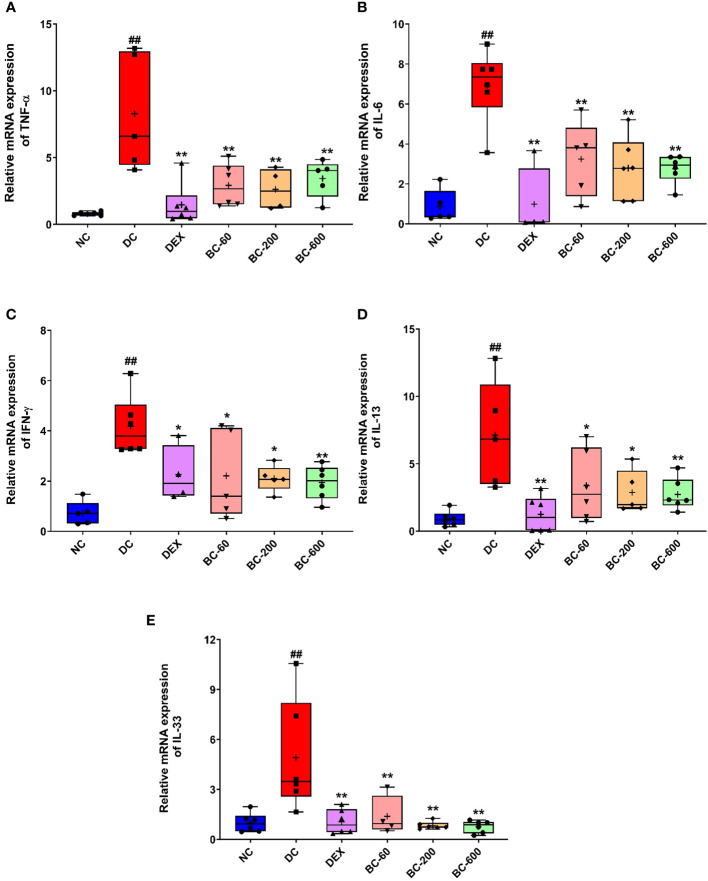
Bronchom reduces the expression of pro-inflammatory cytokines, IL-13 and IL-33 in the lungs. The mRNA expression of the selected cytokines was assessed by employing qRT-PCR. **(A)** TNF-α, **(B)** IL-6 **(C)** IFN-γ **(D)** IL-13 and **(E)** IL-33. Data is presented as mean ± S.E.M (n= 4-6 animals per group). The average value for each of the whisker plots has been denoted by the + symbol. The data was analyzed by one-way ANOVA followed by Dunnett’s multi-comparison *post hoc* test. ##, p < 0.01 vs. saline-challenged (Normal-control) group. *, p < 0.05, **, p < 0.01 vs. disease-control group.

In the current study, we also evaluated the effect of Bronchom on the mRNA expression of pro-inflammatory cytokines (TNF-α, IL-6 and IFN-γ), one Th2 cytokine: IL-13 and in addition, IL-33, another pro-inflammatory cytokine, which is regarded to be central for the generation of Th2-type cytokine-related immune responses. Since, Bronchom at the dose of 20 mg/kg, b.i.d. did not demonstrate significant efficacy in most of the tested parameters, the mRNA expression analysis was conducted in the lungs of the animals that received Bronchom at the doses 60, 200 and 600 mg/kg, b.i.d. HDM-challenge in vehicle-treated animals significantly augmented the TNF-α expression (**Figure 10A**), when compared to the normal-control group. This effect was significantly inhibited by Dexamethasone and Bronchom at the doses of 60, 200 and 600 mg/kg, b.i.d, respectively (**Figure 10A**).

The expression of IL-6 was significantly increased in HDM-challenged animals, as compared to the saline-challenged animals (**Figure 10B**). Dexamethasone significantly inhibited the observed increase (**Figure 10B**). Similarly, Bronchom also significantly attenuated the increased expression of IL-6 at 60, 200 and 600 mg/kg, b.i.d., respectively, **Figure 10B**).

Further, the repeated exposure to HDM in disease-control animals, significantly increased the expression of IFN-γ (**Figure 10C**). This observed elevation was significantly reduced by dexamethasone (**Figure 10C**) as well as by Bronchom at 60, 200 and 600 mg/kg, b.i.d. respectively (**Figure 10C**).

Additionally, the expression of IL-13, another key Th2 cytokine was significantly elevated by HDM-challenge, when compared to saline-challenged group (**Figure 10D**). This increase was significantly suppressed by Dexamethasone (**Figure 10D**) and Bronchom at 60, 200 and 600 mg/kg, b.i.d. respectively, **Figure 10D**).

The mRNA level of IL-33 was also significantly elevated in the disease-control group, as compared to the normal-control group (**Figure 10E**). Dexamethasone significantly reduced the expression of IL-33 (**Figure 10E**). Likewise, Bronchom also attenuated the increase in the expression of the evaluated cytokine at 60, 200 and 600 mg/kg, b.i.d., respectively (**Figure 10E**)

### 3.7 Bronchom regulates HDM-induced alterations in pro- and antioxidant markers in the lungs

**Figure 11:**
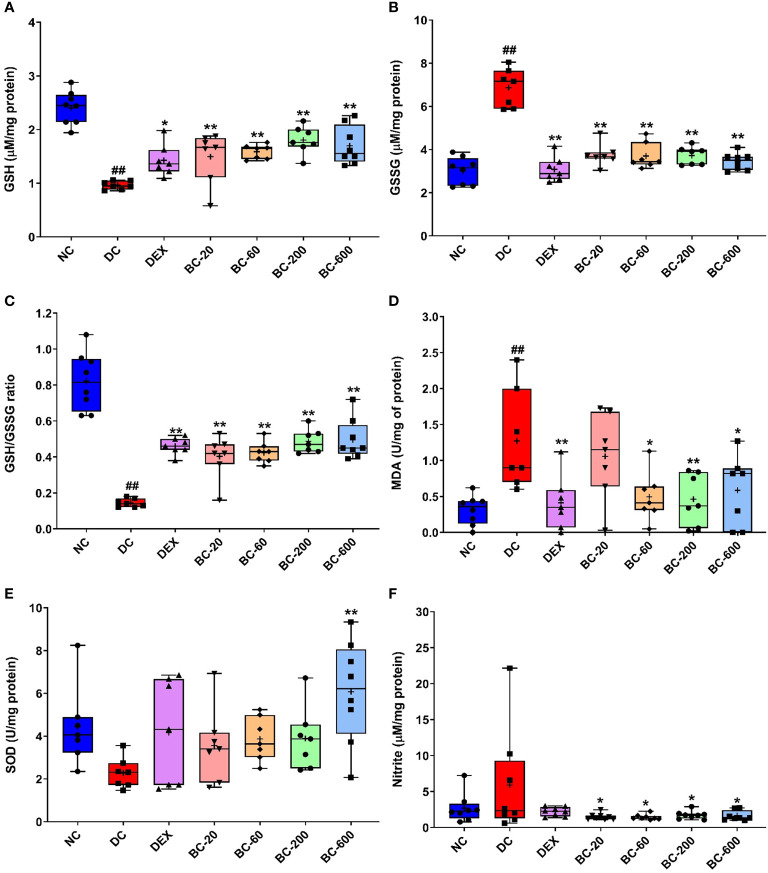
Bronchom alleviates HDM-induced oxidative and nitrosative stress in the lungs. The harvested lungs were subjected to assessment of oxidative and nitrosative stress-related biochemical parameters with the levels and enzyme activities normalized to the protein content estimated in the lung, respectively. **(A)** GSH **(B)** GSSG **(C)** GSH: GSG ratio **(D)** MDA **(E)** SOD **(F)** Nitrite. Data is presented as Mean ± S.E.M (n=7-8 animals per group). The average value for each of the whisker plots has been denoted by the + symbol. and was analyzed by one-way ANOVA followed by Dunnett’s multi-comparison *post hoc* test. ##, p < 0.01 *vs.* saline-challenged (Normal-control) group. *, p < 0.05, **, p < 0.01 *vs*. HDM-challenged (Disease-control group).

When compared to the normal-control group, a significant decrease was noted in the anti-oxidant biomarker, GSH in the HDM-challenged animals (**Figure 11A**) with a concomitant elevation of its oxidized form, GSSG (**Figure 11B**). Further, the ratio of the reduced to oxidized glutathione was also significantly reduced (**Figure 11C**). Reference control drug, dexamethasone significantly restored the levels of GSH (**Figure 11A**) and suppressed the level of GSSG (**Figure 11B**). Additionally, it also reinstated the altered GSH/GSSG ratio (**Figure 11C**). Similar to the dexamethasone, oral administration of Bronchom, significantly restored the levels of GSH at all the tested doses (**Figure 11A**); decreased the level of GSSG at all the tested doses (**Figure 11B**) and furthermore, restored the decrease in GSH/GSSG ratio that was noted in the vehicle-treated, HDM-challenged group (**Figure 11C**).

Furthermore, we also evaluated the effect of Bronchom on MDA activity, a marker of lipid peroxidation in the lung homogenate. The MDA activity was significantly elevated in HDM-challenged animals, when compared to the normal-control group (**Figure 11D**). Both dexamethasone and Bronchom were able to inhibit the HDM-induced increase in MDA activity. The effects were significant for dexamethasone-1 mg/kg, q.d., whereas Bronchom demonstrated significant inhibition at the doses of 60, 200 and 600 b.i.d., respectively (**Figure 11D**).

HDM-challenged animals also exhibited a decrease in SOD activity, which was however not significant when compared to saline-challenged animals (**Figure 11E**). Dexamethasone (1 mg/kg, q.d.) and Bronchom (20, 60 and 200 mg/kg, b.i.d.) both tended to restore the reduced SOD activity. However, Bronchom-600 mg/kg, b.i.d. showed a significant elevation of SOD activity (**Figure 11E**).

Finally, we additionally assessed the nitrosative stress parameter, nitrite in the lung homogenates. HDM-challenge tended to result in an increase in the nitrite levels when compared to the saline-challenged animals (**Figure 11F**). This increase was significantly attenuated by Bronchom at all the tested doses as compared to the disease-control group (**Figure 11F**). Dexamethasone also tended to lower the HDM-induced increase in lung nitrite levels (**Figure 11F**).

## 4 Discussion

Bronchial asthma of the atopic phenotype is the most commonly encountered obstructive airway disease in the clinics. This phenotype is typically characterized by allergen-evoked influx of inflammatory cells in the airways and the resultant airway remodeling, which culminates in symptomatic manifestations such as airway hyperresponsiveness and airway obstruction. The chronic airway inflammation associated with atopic asthma results due to stimulation of both the innate and adaptive immunity. To elaborate, the pathophysiology associated with the Th2 endotype of asthma involves presentation of the antigen to the naïve T-cells by the dendritic cells, which then leads to the genesis of the antigen specific Th2 cells. The allergen-activated Th2 cells secrete several cytokines which orchestrate bronchoconstriction, inflammation and airway remodeling associated with asthma (16).

One of the paramount unaddressed medical need in the pharmacotherapy of asthma is that despite the availability of effective therapies such as inhaled corticosteroids and long acting β_2_-adrenoceptor agonists, approximately one-third of the patients remain uncontrolled or symptomatic or both, which increases the risk for exacerbations and hospitalizations, thereby deteriorating the quality of life of asthma sufferers (6). A common cause for treatment unresponsiveness is sub-optimal adherence to inhaled pharmacotherapy. Medication related factors, unintentional and intentional poor adherence are the main reasons for treatment failure (1). Consequently, newer effective, safe and most importantly oral therapies, which ensure better patient compliance are required. Hence tapping into the traditional medicines, which are derived from natural sources, such as herbs and minerals is a viable, promising and alternative approach towards the treatment of asthma (17).

Bronchom is a calcio-herbal formulation, which has been specifically formulated by encompassing the age-old traditional wisdom of Ayurveda for the treatment of obstructive lung disorders. The sub-acute toxicity of Bronchom has been conducted in both male and female rats as per The Organization for Economic Cooperation and Development (OECD) test guideline number 407 wherein, Bronchom was found to be devoid of any toxicological abnormalities, including in the kidney and liver, up to the highest tested dose of 1000 mg/kg/day, after 28-days repeated dosing (Data under publication elsewhere). In the current study, we evaluated the efficacy of the formulation in mouse model of allergic asthma, which was developed by repeated intranasal instillation of the clinically relevant antigen, house dust mite. It is reported that half of the patients of atopic asthma are sensitized to house dust mites, which implies that HDM is the most common disease-causative allergen (6). More importantly, allergy to HDM is one of the major factors, which may lead to development of severe treatment-resistant asthma (18). The various body parts of house dust mites, their feces, cuticles and eggs can trigger clinical allergy and more than 20 potential allergens have been recognized from HDM. Most of them are either cysteine or serine proteases which are supposedly recognized by several host receptors including Toll-like receptors, C-type lectin receptors, retinoic acid-inducible gene 1-like receptors, NOD-like receptors and AIM2-like receptors. Subsequent to allergen recognition, loss of epithelial integrity occurs leading to cytokine-driven inflammation, which culminates in the manifestation of asthma (19). The results of the present study conclusively demonstrate that all the cardinal features of allergic asthma developed successfully in the model. These observations are in agreement with previously conducted studies, which have employed HDM as a sensitizing allergen (20, 21). In the current experiment, we chose to evaluate the pharmacological effects of Bronchom in female mice, on the basis of a previously published comprehensive study, which solely characterized the development of HDM induced-chronic airway inflammation and airway remodeling in mice (8). Furthermore, previously reported studies have established that female mice are more prone for the development of chronic asthma, subsequent to challenge with HDM, largely due to Th2 cytokine release and humoral response, resulting in higher numbers of eosinophils and more responsiveness to methacholine, when compared to male animals (22, 23). The test article, Bronchom demonstrated promising efficacy by reducing the development of HDM-induced functional, cytological, histopathological, biochemical and molecular alterations.

Airway hyperresponsiveness is one of the main characteristic features associated with bronchial asthma can be defined as an increased bronchoconstrictor response to non-specific agonists in asthma patients. AHR is thought to result from the remodeling changes of the airways like inflammation, increased bronchial smooth muscle mass, increased mucus secretion and sub-epithelial fibrosis (24, 25). In the present study, Bronchom dose-dependently decreased the development of airway hyperresponsiveness to aerosolized methacholine in mice. Interestingly, the percentage protection elicited by the highest dose of Bronchom was not significantly different from that demonstrated by the highly potent oral steroid, dexamethasone. Although, this study was not specifically designed to compare the effects of oral steroids and Bronchom, nonetheless the post-hoc secondary outcome of the study does suggest that the effect of Bronchom’s highest dose seems comparable to dexamethasone in limiting the development of AHR in mice, when challenged with methacholine at a concentration of 50 mg/mL. It is being long known that eosinophils are the major pro-inflammatory cells involved in the pathogenesis of asthma. Eosinophils directly damage the respiratory epithelium due to the release of eosinophil major basic protein and eosinophil cationic protein, thereby contributing to the development of airway hyperresponsiveness (26). More recently, it has been categorically proven that patients demonstrating high sputum eosinophilia are associated with severe and difficult to treat asthma phenotypes (27). Consequently, the potent and dose-dependent reduction of HDM-induced BALF eosinophilia elicited by Bronchom may explain in part the observed prevention of AHR in mice. Furthermore, the data also suggests the preclinical potential of Bronchom to limit exacerbations in severe asthmatics. In addition, since allergic asthma is predominantly a Th2 lymphocyte-driven disease, the decrease in HDM-induced BALF lymphocytosis by Bronchom is an encouraging finding, as a marked reduction will eventually lead to lowering of Th2-associated cytokines as well. As opposed to the Th2 endotype, neutrophils are characteristically associated with non-allergic asthma (1). However, neutrophil influx is observed in allergic asthma as well, immediately after allergen challenge and during virus-induced exacerbations (28). In the present study, we did observe BALF neutrophila, which was albeit lesser than the observed eosinophilia and lymphocytosis in the BALF, which is in agreement with the previously reported studies (21, 29). The observed decrease in BALF neutrophilia elicited by Bronchom suggests that it possesses potential for treating mixed granulocytic and non-Th2 asthma as well, which needs to be evaluated in future studies. In addition to the BALF, inflammatory cell infiltration was also observed in the lung tissue, which was reduced by Bronchom in a dose-related manner, implying that Bronchom demonstrates potential for ameliorating the inflammation-associated changes in the airways as well.

Mucus hypersecretion is commonly seen in asthmatics and formation of relatively solidified mucus plugs is observed in patients with severe asthma (30). Excessive mucus contributes to asthma-associated airway obstruction and AHR. The histological anomaly responsible for increased mucus secretion is goblet cell metaplasia, which results due to the epithelial-mesenchymal transformation and in response to epithelial cell injury, which is orchestrated by pro-inflammatory and Th2 cytokines (31). In the present study, as expected, HDM challenge led to induction of goblet cell metaplasia, which was dose-dependently reduced by Bronchom, suggesting that the calcio-herbal formulation has potential for reducing mucus hypersecretion as well as the prevention of the formation of mucus plugs.

Another airway remodeling characteristic associated with asthma is the development of fibrosis in the sub-epithelial regions of the bronchi as well as the around the blood vessels. This histological emergence can lead to fixed airflow limitation and it develops subsequent to epithelial injury-induced activation of the epithelial-mesenchymal trophic unit (32). Eosinophil derived growth factors and Th2 cytokines mediate the development of sub-epithelial fibrosis (33). HDM-challenge in the present study also led to sub-epithelial fibrosis and its appearance was notably decreased by Bronchom.

Increased airway smooth muscle (ASM) mass, surrounding the bronchi and bronchioles is clinically observed in asthmatics and it also contributes to obstructive airway symptoms including AHR, as a direct consequence of hypercontractility (34). With an objective of quantifying the increase in ASM mass, immunofluorescent staining for α-smooth muscle actin (α-SMA) was performed in the current experiment. α-SMA is expressed in smooth muscle cells and myofibroblasts and can hence be employed as a marker to assess the changes in ASM (35). Moreover, exposure to HDM in experimental animals can also lead to increased α-SMA (21). Similar to the development of goblet cell metaplasia, increased expression of α-smooth muscle actin is coordinated by Th2 cytokines and growth factors (36). In agreement with the reported literature, increased α-SMA immunofluorescent staining was also successfully established in the current study and was reduced by Bronchom in a dose-related fashion. Taken together, the histological findings convincingly demonstrate that Bronchom reduced inflammatory cell influx in BALF and airways and additionally ameliorated goblet cell metaplasia, sub-epithelial fibrosis and increase in ASM which explains the protection of mice from developing AHR subsequent to HDM challenge.

Since the observed airway remodeling changes evoked by HDM is mediated by Th2 cytokines, proinflammatory cytokines and chemokines and Bronchom demonstrated efficacy in ameliorating the observed HDM-induced cytological and histological changes as well as the functional readout of AHR, the current study also aimed to evaluate the effect of Bronchom on these inflammatory mediators. At a biochemical level, Bronchom decreased the HDM-induced release of Th2 cytokines, namely IL-4 and IL-5 as well as the chemokines, eotaxin and IP-10 in the BALF. In addition, Bronchom reduced the mRNA expression of TNF-α, IL-6, IFN-γ and IL-33, which are pro-inflammatory cytokines as well as IL-13, which is a Th2 cytokine in the lung. The role of IL-4, IL-5, IL-6, IL-33, TNF-α and IFN-γ in the pathogenesis of allergic asthma is well documented has been elaborately explicated elsewhere (17, 21, 37). Eotaxin, a CC chemokine, is a potent eosinophil chemoattractant, which mediates the chemotaxis of eosinophils from the blood vessels present in the lung into the BALF and later the airways by acting on the CCR3 receptor present on the surface of the eosinophil (38). Moreover, in experimental models, repeated HDM challenge also leads to increase release of eotaxin in the BALF (21). Given the pivotal significance of eosinophils in Th2 asthma endotype, eotaxin holds a central role in mediating the pathogenesis of asthma. Bronchom, in the present study reduced the BALF levels of eotaxin, which explains in part, the attenuated HDM-induced eosinophilia reported in the current study. On the other hand, IP-10 is a CXC chemokine, which is responsible for the chemotaxis of cytotoxic T-cells and natural killer cells, especially during viral infections, a common reason for exacerbations of asthma. IP-10 leads to lymphocyte migration primarily by acting on the CXCR3 receptors present on the surface of the leukocytes (39). Nevertheless, increased IP-10 release has also been reported subsequent to HDM-challenge in mice (21) and we also observed increased levels of the CXC chemokine in the present study. Interestingly, Bronchom decreased the HDM-triggered increase of IP-10 in the BALF pointing towards its potential towards preventing viral exacerbations in asthma sufferers.

Although oxidative stress is unequivocally associated with the pathogenesis of chronic obstructive pulmonary disease, reactive oxygen and nitrogen species contribute to the development of inflammation, AHR, lung injury and the resultant airway remodeling in bronchial asthma as well. The balance of endogenous oxidants and antioxidants is thought to be impaired as a result of oxidative and nitrosative stress observed in asthma. To exemplify, the activity of the anti-oxidant superoxide dismutase (SOD) is decreased in asthmatics and it contributes to airway inflammation and AHR (40). Similarly, nitrite concentration, a marker of nitrosative stress is reported to be increased in the exhaled breath in atopic asthmatics with exacerbations and is therefore considered to be a suitable marker of nitrosative stress (41). The role of oxidative stress in the pathogenesis of asthma has been elucidated in detail in previous studies conducted in our laboratory (17, 37). In the context of present study, oxidative stress has also been reported subsequent to HDM-sensitization and challenge in mice, wherein activities of the constitutive antioxidants like SOD, catalase and glutathione peroxidase were decreased. γ-tocotrienol, an isoform of vitamin E demonstrated free radical scavenging activity and as a result downregulated HDM-induced features of allergic asthma (42). In another study, decreased levels of reduced glutathione and consequently increased lipid peroxidation have also been reported in mice exposed to HDM (43). In the current study we observed significantly decreased reduced glutathione and increased oxidized glutathione in the lungs of HDM-challenged animals, along with a significant increase in the levels of malondialdehye, a marker of lipid peroxidation. Similarly, a trend towards reduced SOD activity and increased nitrite levels could also be detected. The calcio-herbal formulation was able to restore the endogenous oxidant/antioxidant balance, which was impaired by HDM-challenge.

Previous studies conducted at our institute have demonstrated promising preclinical efficacy of two calcio-herbal formulations, namely Divya Swasari Ras and Divya Swasari Kwath in mouse models of allergic asthma induced by ovalbumin (17, 37). Bronchom is a newly developed Ayurvedic formulation, which contains extracts of fifteen and dry powders of nine medicinal plants. Additionally, it is also supplemented with four herbally processed mineral preparations referred to as ‘Bhasmas’ in Ayurveda (**Table 1**). The components of Bronchom have been traditionally used for lung disorders. UHPLC-PDA analysis of Bronchom detected the presence of eight bioactive phytometabolites namely, gallic acid, protocatechuic acid, methyl gallate, rosmarinic acid, glycyrrhizin, eugenol, 6-gingerol and piperine. Of these components, rosmarinic acid in Perilla extract has demonstrated efficacy in HDM-induced allergic asthma (44). In this study reported by Sanbongi et al, 2004, mice received 1.5 mg of the Perilla extract per day, which contained 68% rosmarinic acid. Consequently, the dose of rosmarinic acid, which the mice would have received per day is computed to be ~1 mg (44). Quantification of the phytocompounds present in Bronchom in the present study revealed that each mg of Bronchom contains 0.793 µg of rosmarinic acid (**Table 2**). The pharmacological effects of Bronchom were majorly evident at 200 and 600 mg/kg, b.i.d., which corresponds to 400 and 1200 mg/kg/day. Considering the daily dose of Bronchom, mice in the present study would have received ~ 8 and ~ 24 µg rosmarinic acid, respectively. These findings suggest that other phytometabolites of Bronchom also contribute to the observed pharmacological effects. Further, as reported by Hu et al., 2022 (45), the in-vivo efficacy demonstrated by acupoint sticking therapy in mouse model of HDM-induced allergic asthma, may in part be attributed to the presence of gingerol. Furthermore, in an animal model of HDM-induced atopic dermatitis, the water extract of *Alpinia officinarum* exerted therapeutic effects which may be associated with the presence of protocatechuic acid (46). Finally, some of the other phytocompounds detected in Bronchom have demonstrated anti-inflammatory, anti-oxidant and anti-allergic activity, which is discussed in detail in our previously published research article (17).

The present study has conclusively established the efficacy of Bronchom in reducing the development of the cardinal features of allergic asthma, induced by the most common clinical allergen, house dust mite. Bronchom diminished AHR, influx of inflammatory cells in BALF and their infiltration in the lung, along with remodeling changes such as sub-epithelial fibrosis, goblet cell metaplasia and increased ASM mass. The plausible mechanism of action of Bronchom is through moderation of Th2 and pro-inflammatory cytokines, chemokines and oxidative stress. Hence, Bronchom possesses a clinicotherapeutic potential in the management of allergic asthma. This is the first report of the evaluation of the preclinical efficacy of Bronchom, in a clinically relevant animal model of atopic asthma. For the proof of concept, Bronchom was administered prophylactically in the current study. Based on the encouraging results in the present study future studies are warranted to evaluate the preclinical effectiveness of Bronchom administered concurrently or therapeutically. These future directed studies would add more body of evidence into the pre-clinical efficacy of Bronchom.

## Data availability statement

The raw data supporting the conclusions of this article will be made available by the authors, without undue reservation.

## Ethics statement

The animal study was approved by Institutional Animal Ethics Committee of Patanjali Research Foundation. The study was conducted in accordance with the local legislation and institutional requirements.

## Author contributions

AB: Conceptualization, Supervision, Visualization, Writing – review & editing. SS: Conceptualization, Data curation, Formal analysis, Investigation, Methodology, Supervision, Visualization, Writing – original draft. SK: Formal analysis, Investigation, Methodology, Writing – original draft. MM: Formal analysis, Investigation, Methodology, Writing – original draft. RD: Data curation, Supervision, Visualization, Writing – review & editing. AV: Conceptualization, Project administration, Supervision, Visualization, Writing – review & editing.
