# Supplementary material for: Bronchom assuages airway hyperresponsiveness in house dust mite-induced mouse model of allergic asthma and moderates goblet cell metaplasia, sub-epithelial fibrosis along with changes in Th2 cytokines and chemokines
Source: Front Immunol. 2024 May 14;15:1384697. doi: 10.3389/fimmu.2024.1384697 (PMC11130375; doi:10.3389/fimmu.2024.1384697)
Supplement: Supplementary file 1 [file Table_1.docx]

**SUPPLEMENTARY TABLE 1** Sequences of the forward (FW) and reverse (RV) primers used for qRT-PCR

| **S. No.** | **Gene Name** | **Primer sequence** |
| --- | --- | --- |
| 1 | TNF-α FW | ATAGCTCCCAGAAAAGCAAGC |
| 2 | TNF-α RV | CACCCCGAAGTTCAGRAGACA |
| 3 | IL-6 FW | TGGAGTCACAGAAGGAGTGGCTAAG |
| 4 | IL-6 RV | TCTGACCACAGTGAGGAATGTCCAC |
| 5 | IL-13 FW | CCTGGCTCTTGCTTGCCTT |
| 6 | IL-13 RV | GGTCTTGTGTGATGTTGCTCA |
| 7 | IL-33 FW | TCCAACTCCAAGATTTCCCCG |
| 8 | IL-33 RV | CATGCAGTAGACATGGCAGAA |
| 9 | GAPDH FW | AAGGTCATCCCAGAGCTGAA |
| 10 | GAPDH RV | CTGCTTCACCACCTTCTTGA |
